# Integrated Natural Language Processing and Machine Learning Models for Standardizing Radiotherapy Structure Names

**DOI:** 10.3390/healthcare8020120

**Published:** 2020-04-30

**Authors:** Khajamoinuddin Syed, William Sleeman IV, Kevin Ivey, Michael Hagan, Jatinder Palta, Rishabh Kapoor, Preetam Ghosh

**Affiliations:** 1 Department of Computer Science, Virginia Commonwealth University, Richmond, VA 23284, USA; william.sleemaniv@vcuhealth.org (W.S.I.); pghosh@vcu.edu (P.G.); 2 Department of Radiation Oncology, Virginia Commonwealth University, Richmond, VA 23298, USA; michael.hagan@va.gov (M.H.); jatinder.palta@vcuhealth.org (J.P.); rishabh.kapoor@vcuhealth.org (R.K.); 3 Department of Computer Science, University of Virginia, Charlottesville, VA 22904, USA; ki4km@virginia.edu; 4 National Radiation Oncology Program, Department of Veteran Affairs, Richmond, VA 23249, USA

**Keywords:** radiotherapy structure names, nomenclature standardization, quality assurance, machine learning, natural language processing, text categorization, TG-263

## Abstract

The lack of standardized structure names in radiotherapy (RT) data limits interoperability, data sharing, and the ability to perform big data analysis. To standardize radiotherapy structure names, we developed an integrated natural language processing (NLP) and machine learning (ML) based system that can map the physician-given structure names to American Association of Physicists in Medicine (AAPM) Task Group 263 (TG-263) standard names. The dataset consist of 794 prostate and 754 lung cancer patients across the 40 different radiation therapy centers managed by the Veterans Health Administration (VA). Additionally, data from the Radiation Oncology department at Virginia Commonwealth University (VCU) was collected to serve as a test set. Domain experts identified as anatomically significant nine prostate and ten lung organs-at-risk (OAR) structures and manually labeled them according to the TG-263 standards, and remaining structures were labeled as Non_OAR. We experimented with six different classification algorithms and three feature vector methods, and the final model was built with fastText algorithm. Multiple validation techniques are used to assess the robustness of the proposed methodology. The macro-averaged F_1_ score was used as the main evaluation metric. The model achieved an F_1_ score of 0.97 on prostate structures and 0.99 for lung structures from the VA dataset. The model also performed well on the test (VCU) dataset, achieving an F_1_ score of 0.93 for prostate structures and 0.95 on lung structures. In this work, we demonstrate that NLP and ML based approaches can used to standardize the physician-given RT structure names with high fidelity. This standardization can help with big data analytics in the radiation therapy domain using population-derived datasets, including standardization of the treatment planning process, clinical decision support systems, treatment quality improvement programs, and hypothesis-driven clinical research.

## 1. Introduction

**Figure 1:**
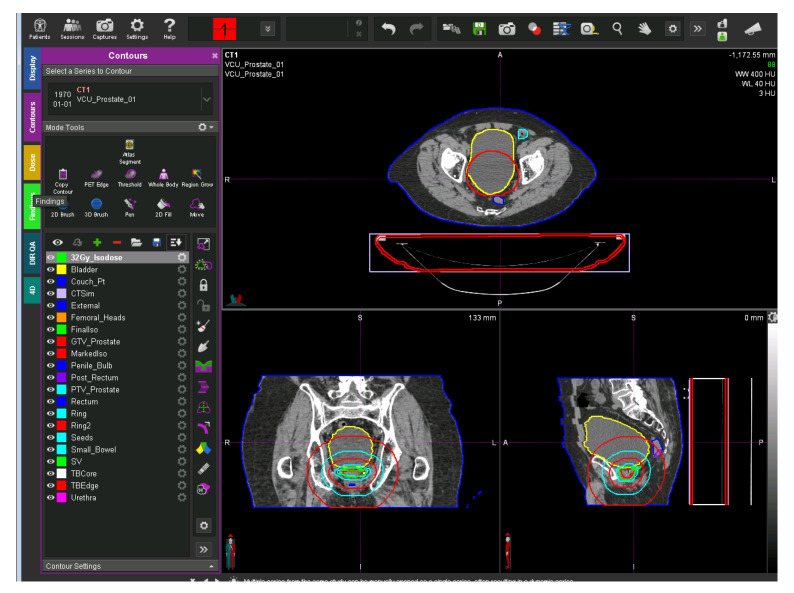
A representative CT image overlaid with its defined structures. The left side of the figure shows the physician-transcribed names of the structures delineated on the right side. The physician-transcribed names and structures delineated can be matched by the color.

Radiation therapy is a type of cancer treatment that uses high intensity energy beams to kill cancer cells and shrink the tumor. In order to treat cancer, the radiation oncologist delineates the tumorous region or target volume on a computed tomography (CT) or magnetic resonance imaging (MRI) dataset. Additionally, the normal organs, known as organs-at-risk (OAR) volumes are delineated to spare and estimate radiation doses and reduce possible side effects. These delineated volumes are known as structures. Radiation oncology team members, such as radiation physicists and dosimetrists, delineate other types of structures termed as “planning organs at risk volume” (PRV). These structures are used strictly in the treatment planning process and take into account the mobility of the organs at risk, and therefore, a surrounding margin is added to these structures to compensate for geometric uncertainties. All delineated structures are given names that are usually written in free text as identifiers, but the lack of standardized nomenclature has created inconsistencies in naming the structures. Figure 1 shows a representative CT image overlaid with its defined structures. The left side of the figure shows the physician-transcribed names of the structures delineated on the right side.

The use of standard nomenclature is an essential step for the construction and use of informatics-based tools to automatically extract pertinent data from electronic medical records in support of clinical trials, data-pooling initiatives, and clinical practice improvement. It also provides a foundation for the development of software tools to automate data extraction, analysis, data submission, exchange, and quality assurance (QA) [1,2].

To address these issues, the American Association of Physicists in Medicine (AAPM) has released a Task Group 263 (TG-263) report with the standardized nomenclature for structures names [3]. This report was developed in collaboration with stakeholders from both multi-institutional and multi-vendor organizations. The American Society for Radiation Oncology (ASTRO) and AAPM have identified the following as the main challenges in RT structure name standardization [3]:

- Vendor-based challenges that originate from the inter-vendor variation on software architecture. Each vendor has a particular character set for naming the structures; limited allowable character sets, however, hinder the interoperability.
- Multi-institutional-based challenges that may arise from the lack of participation, oversight, and guidelines in creating a standardized nomenclature.
- Single institutional challenges include data governance issues; costs; and difficulties of implementing new nomenclatures, making them compatible with existing treatment modalities, and training the institutional staff to follow the standards.
- Clinical staff challenges may encompass the lack of guidelines or a detailed schema to follow.

Strict adherence to a standardized nomenclature will help to achieve future standardization, but it cannot address retrospective data standardization. Manually relabeling inconsistent names with the corresponding standardized TG-263 names is one way to correct retrospective data; however, generating such mappings for multi-center data is slow, time consuming, inefficient, hard to generalize, and challenging to scale. This sets the stage for machine-learning-based methods that may be able to overcome some of these limitations. To address each of the issues mentioned above, we propose a methodology to retrospectively standardize the radiotherapy structure names using a combination of machine learning and natural language processing techniques.

The main contributions of this paper are:

- Proposing a novel automated machine learning approach to standardize the physician-given structure names to the domain wide utilized TG-263 standard names.
- Demonstrating that a relatively small amount of data from each center is enough to build a generalizable machine learning model, which a simple text mapping cannot achieve.
- Establishing that the approach is disease site agnostic; it can be used on multiple disease sites.
- Demonstrating that physician-given names hold enough information about the structures that can be utilized to predict the standard name.
- Creating a scalable approach that requires little to no preprocessing.

## 2. Related Work

The existing techniques for structure name standardization can be broadly classified into three categories: expert-based, ontology-based, and machine-learning-based.

Previous works in the RT community to retrospectively standardize structure names mostly use the physician provided names (free-text labels) or geometric information such as volume, area, and location of the structures. The recently published works to standardize structure names using physician-given names are illustrated as below.

A research team in Australia recently proposed an expert-based approach to standardize radiotherapy structure names as per the TG-263 standard recommendations [4]. In this study, a panel of experts developed a mapping and structure synonym set for 36 structures from their clinical database. With their method, they were able to map 99% of the relevant structures and relabel the names correctly. However, the major limitation of this approach are scalability and generalizability; data used in this project were from a single academically focused institution that could enforce the local standards, and the mappings were dependent on inputs provided by experts. This method is also center specific; mappings from one institute may not be useful to the other institute.

A different team in the Netherlands has proposed an ontology-based RESTful web service to standardize the structure names [5]. However, this approach was more focused on building a linked data than a technique for structure name standardization. The authors used the mappings provided by the institutions to generate centralized mappings, thereby creating a common terminology for linked data.

There are few papers that have proposed machine-learning-based approaches to structure name standardization. Unlike expert-based and ontology-based methods, machine-learning-based methods use either free text labels or geometric information to build learning models for standardization. One such work made use of multiple string similarity measures to generate feature vectors, and these feature vectors were used as input for the classification algorithm to predict the labels [6]. This paper used neural-network-based methods but lacked the pertinent details for reproducibility of the results. Two other papers proposed methods using geometrical information for structure name standardization [7,8]. Both of these papers have used a machine learning approach with neural networks to standardize the structure names of the head and neck region. Even though they both showed a high accuracy for identifying the standard names, the major limitation of these works was that they considered only limited OAR structures to build the ML model and Non_OARs were discarded. Removing Non_OAR structures makes it difficult to apply these two approaches in the real-world datasets because real-world datasets will contain both OARs and Non_OARs.

Expert-based methods have high accuracy but require manual effort from experienced clinicians, which makes scalability and generalizability challenging to achieve. Although ontology-based techniques can help in automating the labeling task, there is a paucity of domain-specific comprehensive ontologies in the radiation oncology. Machine-learning-based methods are well suited for retrospective structure name relabeling but are seldom used in this domain. Additionally, the TG-263 standardization was only completed in 2018 [3], and hence applications of machine-learning-based methods for structure name prediction are still in their infancy.

## 3. Methods and Materials

### 3.1. Data

Across the United States, the Veterans Health Administration (VA) has 40 centers treating veterans with in-house radiation therapy services. The VA has put together the Radiation Oncology Quality Surveillance Program (VA-ROQS), and as part of this program the treatment quality is assessed from all VA centers [9]. As part of the initial pilot study, data from all 40 centers were manually abstracted from clinical charts, imaging databases, and radiation oncology specific systems, such as treatment planning systems and treatment management systems. Data from up to 20 prostate and 20 lung cancer patients were manually abstracted from each center, resulting in a total of 794 and 754 patients respectively. The collected data included the DICOM (Digital Imaging and Communication in Medicine) structure set files representing anatomical structures of interest and the corresponding DICOM CT image datasets for each patient. For this project, ten lung and nine prostate OAR structures were identified. These structures were manually labeled to their TG-263 standard names, and all other structures, including target and PRVs, were labeled as Non_OAR. The dataset will be further referred to as the VA-ROQS dataset.

We also collected data from the Department of Radiation Oncology at Virginia Commonwealth University (VCU) as an external test dataset, which included DICOM structure set data from 50 randomly selected patients with prostate cancer and another 50 patients with lung cancer. The same procedure that was used in the VA-ROQS data preparation was also used to label the structures in this dataset, which will be referred to as the VCU dataset. The following prostate and lung OAR structures considered in this work are:

Prostate organs-at-risk structures: Bladder, Rectum, LargeBowel, SmallBowel, Femur_L, Femur_R, SeminalVesicles, PenileBulb, and External.

Lung organs-at-risk structures: Heart, Esophagus, Lungs, Lung_R, Lung_L, SpincalCord, BrachialPlexus, BrachialPlexus_L, BrachialPlexus_R, and External.

Table 1 shows the distributions of lung structures for the VA-ROQS and VCU datasets, while Table 2 shows the distributions of the prostate structures in these two datasets. In both cases, the Non_OAR structures present an overwhelming majority; these Non_OARs include all the structures contoured as a part of treatment planning and delivery and the dose evaluation structures. We also observed similar class imbalances across all VA-ROQS centers’ data (see Figures S1 and S2 in the Supplementary Material). Table 3 shows the examples of physician-given names compared to the standard OAR structures, which highlights the variability in the physician-given names. Table 1 also shows the number of unique names found in each Lung structure in the VA-ROQS and VCU datasets, and Table 2 shows physician-given unique names for the prostate structures in VA-ROQS and VCU datasets.

### 3.2. Data Preprocessing

Structure names are short and have a limited character set to use, and the available character set is vendor dependent. As shown in Table 3, even though there is high variability in physician-given structure names for most of the structure type, the character set used is limited. Preprocessing methods need to be selected to ensure that critical information is retained; losing the information might negatively affect the ability to standardize the structure names with high fidelity. Hence, we decided to keep the preprocessing of physician-given names to a minimum by just converting them to lower case.

### 3.3. Model Selection

After preprocessing the data, the next step is to select the appropriate machine learning method. We experimented with different types of methods to map the physician-given structure names to the TG-263 standardized names. The datasets presented have some unique characteristics that impacted the choices and performances of our algorithms. Structure names are very short in size (varying between 4 and 20 characters), which limits the use of complex machine learning algorithms [10]. For better applicability of the machine learning algorithms, we identified the features from the structure names to build the feature vectors, which are necessary for any machine learning algorithm.

Since machine learning algorithms work on numerical data, we converted the text data into numerical features. Numericalization of text data involves two steps [11]: (1) tokenization or feature set generation and (2) vectorizing the features with feature weight calculation techniques. We tried multiple feature generation and feature weight calculation methods, as discussed next.

We tested the following list of techniques for feature set generation.

1. Bag-of-words (BoW): In this model, text (such as a sentence or a document) is represented as the bag (multiset) of its words, disregarding grammar and even word order but keeping multiplicity [12]. The bag-of-words model has also been used extensively in the natural language processing domain. For example, bag-of-words features for the physician-given name "femoral head left" are "femoral," "head," and "left."
2. Word n-grams: An n-gram is a contiguous sequence of n terms from a given sequence of text. Given a sentence, we can construct a list of n-grams from it by finding pairs of words that occur next to each other. For example, with a physician-given name, "femoral head left," we can construct bigrams (n-grams of length 2) by finding consecutive pairs of words; "femoral head" and "head left" are bi-grams.
3. Character n-gram: In this model, instead of considering a full token or a term, a set of continuously occurring characters is used to build the feature set. These character sets are considered to form n-gram features. For example: with the physician-given name "bladder," character three-gram features are "bla," "lad," "add," "dde," "der."

Assigning appropriate weights to individual features as per their relevance in a given dataset is known as feature weighting. It is generally thought of as a generalization of feature selection, where the presence of a feature serves as the criterion for its extraction. We used various feature weighting methods to build the feature vectors, as shown below.

1. Term presence (tp): In this method the presence or absence of a term in the given document is encoded as 1 or 0.
2. Term count (tc): This method is an extension of the tp method. Here, term occurrence is considered as the weight; it denotes the number of times a given term appears in a document.
3. Term frequency (tf): In this method, the term occurrence is usually normalized to prevent a bias towards longer documents (which may have a higher term count regardless of the actual importance of that term in the document) from giving a measure of the importance of the term t within the particular document d. Thus we have the term frequency, defined as follows [13,14].
  (1) $${tf}_{t,d}=1+\log{tf}_{t,d}$$
4. Term frequency-inverse document frequency (tf-idf): tf-idf is a numerical statistic that reflects how important a word is to a document in a collection or corpus [15]. It involves two parts: First is tf, which is defined as in Equation (1). Second is inverse document frequency (idf)), which is a measure of the general importance of the term (obtained by dividing the total number of documents by the number of documents containing the term, and then taking the logarithm of that quotient).
  (2) $${\mathrm{idf}}_{t}=\log\frac{N}{{\mathrm{df}}_{t}}$$
  (3) $${\mathrm{tf}\text{-}\mathrm{idf}}_{t,d}={\mathrm{tf}}_{t,d}\cdot {\mathrm{idf}}_{t}$$
  In Equations (1)–(3), tf is term frequency, df is document frequency, t is term, d is document, ${df}_{t}$ is number of documents a term (t) appears in, and N is the total number of documents.
5. Word embeddings: Words or phrases from the vocabulary are mapped to vectors of real numbers. Conceptually, it involves a mathematical embedding from a space with many dimensions per word to a continuous vector space with a much lower dimension; word2vec [16], Glove [17], and fastText [18] are some of the well known word embedding techniques.

#### Feature Weighting Example

Here we show the examples of each of these weighting methods. Consider four physician-given names: (1) *large bowel*, (2) *sigmoid colon*, (3) *bowel*, and (4) *bowel lg*. If we consider the bag-of-words model for feature set generation, our feature set will consist of unique tokens from the above mentioned four names, which are { large, bowel, sigmoid, colon, lg }. The total number of documents is four (*N* = 4) (physician-given names). Below are feature vectors with each of the weighting methods for physician-given name “large bowel” as below.

$$\begin{array}{c} feature\_Set=\left[\begin{array}{c} \;large\;bowel\;sigmoid\;colon\;lg\; \end{array}\right]\\ tp=\left[\begin{array}{c} \;1\;1\;0\;0\;0\; \end{array}\right]\\ tc=\left[\begin{array}{c} \;1\;1\;0\;0\;0\; \end{array}\right]\\ tf=\left[\begin{array}{c} \;0.5\;0.5\;0\;0\;0\; \end{array}\right]\\ tf-idf=\left[\begin{array}{c} \;1.301\;0.087\;0\;0\;0\; \end{array}\right] \end{array}$$

We used six different classification algorithms—SVM-linear [19], SVM-RBF [20], k-nearest neighbors (KNN) [21], logistic regression [22], random forest [23], and fastText [18]—for initial model selection. All models were built by using scikit-learn machine learning library in python [24]. The best model was selected based on their performance on the VA-ROQS dataset. Tables S1 and S2 show the performances of these models for the different feature vector methods. One of the objectives of this work was to understand the impact of feature weighting techniques on model performance. A thorough comparison of feature weighting techniques and their effects on structure name standardization is beyond the scope of this study. Nevertheless, we report the observations we made during the initial model selection as below.

Tables S1 and S2 show the machine learning model performance with different feature weighting methods. We observed that the tp, tc, and tf with all combinations of ML algorithms produced the same results. We believe these three feature weighting techniques produce the same feature vectors, where tp and tc produce the same vector, and tf is a normalized version of the tc. We believe this is because of the unique characteristics of our dataset. Instances (physician-given names) are short, and words within the names are not repeated. The examples shown above indicate the same. As we know from Equation (3), the tf-idf feature weighting technique takes the global picture of words into account in the calculations, which changes the weights of the features when compared to other methods. Interestingly, tf-idf did not perform well when compared to the other weighting methods for both prostate and lung disease datasets. In comparison with all weighting methods, the word vector based fastText algorithm consistently outperformed all other algorithms; hence we selected it to build our final model.

### 3.4. Model Evaluation

An essential part of building a machine learning system is to demonstrate its quantifiable generalizability. For example, the critical goal of a machine learning classification algorithm is to create a learning model that accurately predicts the class labels of unseen data samples. Hence the machine learning model should work well for classifying future data.

Model validation is an important step in the machine learning process. Evaluation of a model on the training dataset would result in a biased score. Therefore the model is evaluated on the held-out set to give an unbiased estimate of model performance. Just a hold-out set validation is not enough to test the robustness and finalize the model. It is recommended to validate the model on the entire dataset [25,26]. One such technique is k-fold cross-validation. To that effect, we validated our models in three different ways on the VA-ROQS dataset and tested it on the VCU dataset (external dataset).

*Model Validation*

1. **70:30**: The VA-ROQS dataset was divided into a 70:30 ratio as the training and validation sets. The split was stratified by TG-263 standard names, which ensured that an equal percentage of data was taken from each standard name for training, validation, and testing, thereby avoiding center-based bias in modeling.
2. ***K*****-fold**: The VA dataset was divided into *K*-folds in such a way that each fold was stratified by standard name. The *K*-1 fold of the data was used for training, and the remaining fold was for validation. This was repeated until all folds were validated. We performed 5-fold and 10-fold cross-validation to better capture the variance in data folds.
3. **Center-based**: The VA-ROQS dataset came from 40 (*n* = 40) different treatment centers. Data from 39 (*n*-1) centers were used for training, and one center’s data was used for testing. We repeated this process until all centers were tested based on the model trained on the remaining *n*-1 centers.

*Model Testing*

Once the model is thoroughly validated and finalized, we need to test it on entirely new data (unseen by the model during training). We built a final model on the VA-ROQS dataset and tested it on the VCU dataset. One of the reasons we choose VA-ROQS for training and VCU for testing was to avoid any overlap of data between the training and test sets.

### 3.5. Evaluation Metrics

The performance of a model can be measured with different evaluation metrics. However, these metrics need to consider the class (structure labels) distribution to evaluate the model accurately. The dataset presented has a high level of class imbalance, as shown in Table 1 and Table 2. Hence we evaluated the performance of each model using four distinct metrics—overall accuracy, macro-averaged precision, recall, and F_1_ score. Overall accuracy simply measures the percentage of OARs in the validation set classified correctly.

A macro-averaged metric computes results for each class independently and then takes the average of all to calculate the overall average metric. In contrast, a micro-average aggregates the contributions of all classes to compute the overall metric. We note that in classification tasks such as ours in which each structure name is mapped to precisely one label, accuracy is the same as the micro-averaged F_1_ score. A micro-averaged F_1_ score and overall accuracy metrics do not disproportionately penalize a classifier for performing poorly on the less frequent classes, whereas macro-averaged F_1_ score is heavily influenced by how well the classifier performs on the less frequent classes. Hence the performance of a rare class and a more frequent class are equally important.

Accuracy measures how well a classifier performs overall, whereas macro-averaged precision, recall, and F_1_ score better capture how well a classifier can identify cases that it does not often see, which is extremely important in real-world settings. The mathematical expressions of each of these metrics are shown below.

(4) $$Precision=\frac{TP}{TP+FP}$$

(5) $$Recall=\frac{TP}{TP+FN}$$

(6) $$F_{1}score=2\cdot \frac{Precision\cdot Recall}{Precision+Recall}$$

(7) $$Accuracy=\frac{TP+TN}{TP+TN+FP+FN}$$

In the above formulae, truth table counts are represented by TP as true positives, TN as true negatives, FP as false positives, and FN as false negatives.

Along with all the above-mentioned metrics, we used a confusion matrix, which is a summary of prediction results on a classification task. The numbers of correct and incorrect predictions are summarized with count values and broken down by each class. The confusion matrix shows how the classification model is confused when it makes predictions. It provides insight not only into the errors made by a classifier, but more importantly, the types of errors that are made. All the metrics mentioned were computed from the confusion matrix.

### 3.6. fastText Classification Algorithm

The fastText text classification algorithm [18] is an extension of the word vector method, which includes three major steps. First, generating the word vectors: fastText learns the vector representation of words from subwords (character n-gram) [27]. For example, the word “Bladder” with a character n-gram of 3 will have fastText representations such as “<bl, bla, lad, add, dde, der, er>” wherein < and > are added to indicate the beginning and end of the word. The technique of breaking the word into character n-gram makes it work well with rare words. This helps to find the vector representation of a word, even if it is not seen in training, and this done by breaking down the word into character n-grams to get the word embedding. A subword size can be selected with range *minn* and *maxn*, indicating the minimum and maximum length of the subwords to generate. Along with these, fastText also considers *wordNgrams* (word n-gram) to build the vector representation. Vector size is selected by setting the *dim* parameter. In Section 3.7 we explained the hyperparameter tuning.

**Figure 2:**
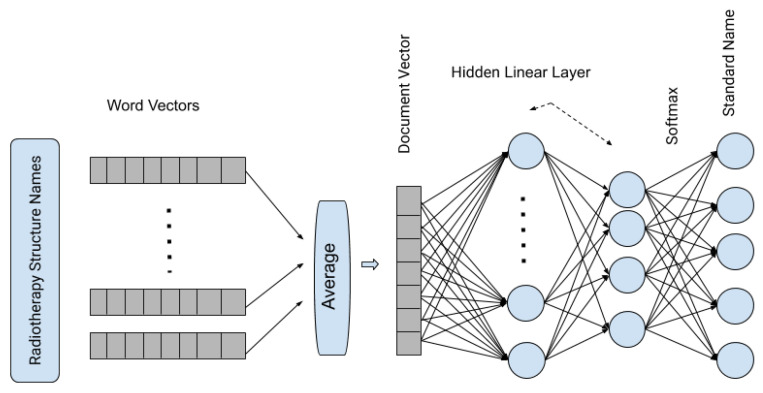
Pictorial representation of fastText supervised classification algorithm.

In the second step, word vectors are averaged to form a document vector, and in our method, it represents the vector representation of the complete RT structure. In the third and final step, it passes the averaged vectors through a shallow neural network with one hidden layer and uses the *softmax* function to generate the probability of a structure is one of the standard RT structures. Figure 2 shows the architecture of the fastText supervised classification algorithm.

### 3.7. fastText Hyperparameter Tuning

After the initial selection of models, we chose fastText for further analysis, as it performed better than all other models. To further improve the model’s performance, selecting appropriate hyperparameter values is important. The fastText algorithm has many hyperparameters, and we chose eight parameters to optimize, which have an impact on the data dictionary and model training. Out of eight hyperparameters selected for model tuning, two hyperparameters *minn* and *wordNgrams* were kept at fixed values. *wordNgrams* selects the number of consecutive individual words while building a data dictionary. Physician-given names are most likely to have less than three distinct words; to avoid considering the complete given name as a token, we set *wordNgrams* to 2. On the other hand, minn provides the minimum number of consecutive characters to consider as a token. We set *minn* to 2 to capture the more meaningful tokens rather than selecting every character as a token. Table 4 shows the hyperparameters and values tested.

A total of 15,360 combinations of hyperparameters was generated; each combination of hyperparameters was used to build a separate model for each disease type, and so considering the two disease types, overall we created 30,720 models. Models were evaluated with metrics described in Section 3.5 on the validation dataset and were recorded separately for each of the diseases types. Figures S3 and S4 show the impact of each hyperparameter on model performance. Boxplots are used to show the distribution of model performance (F_1_ score) for each value of the hyperparameter; the value with the smallest inter-quartile range and highest median was selected. The hyperparameter value was selected based on its performance on both disease type data (prostate and lung). The best values for hyperparamter selected are shown in Table 4 with brief descriptions.

## 4. Results

In this section, we present the results of our models for both the VA and VCU datasets. We built models with combinations of feature sets, feature weighting methods, and machine learning algorithms. We observed that among all models, the fastText model performed consistently well on our data. Hence we present the detailed descriptions of results from only the fastText models. Results from the remaining models are shown in the Supplementary Material. The macro-averaged precision, recall, F_1_ score, and overall accuracy for both prostate and lung datasets for all the validation types are shown in Table 5. Individual class level results are shown in Tables S4–S7 for prostate and Tables S9–S12 for lung in the Supplementary Material.

After fastText was selected as a final model, we tested the robustness of this method with four different validation types. Each of the validation types tested a different aspect of our model performance. Below we describe the results for each of these validation types.

### 4.1. Validation Results

**Figure 3:**
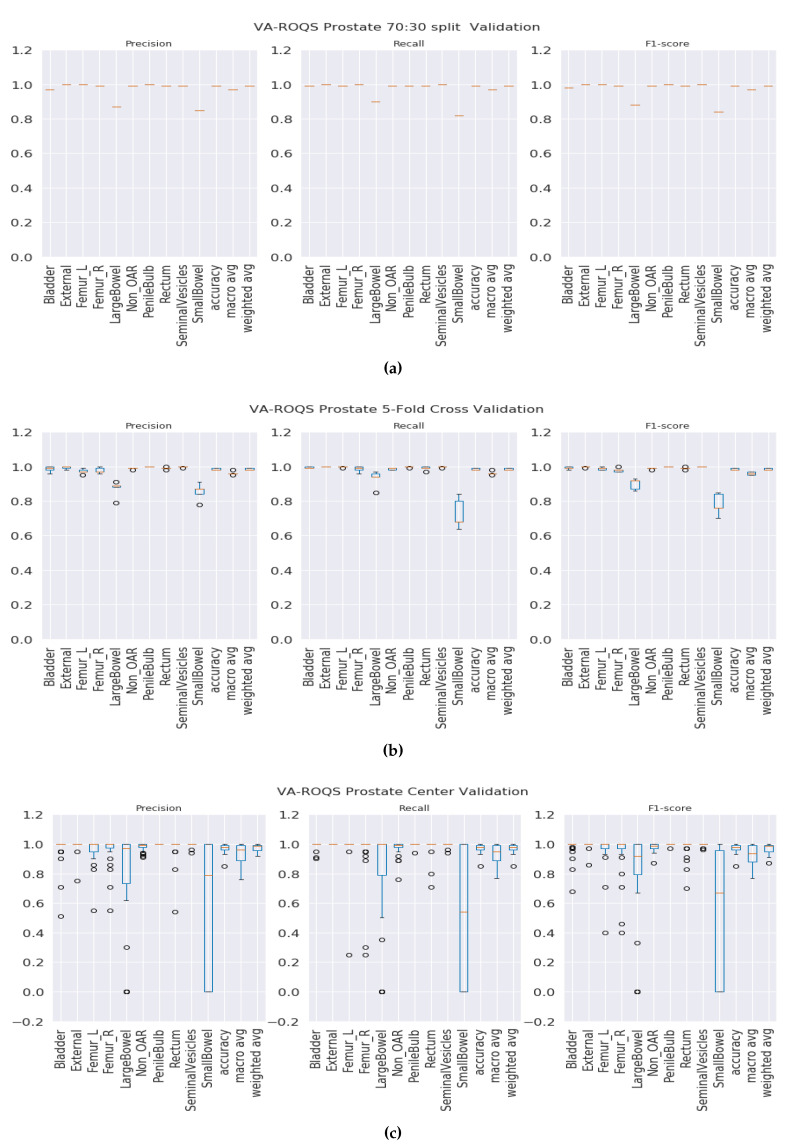
VA-ROQS prostate dataset—cross-validation results: (**a**) VA-ROQS 70:30 split cross-validation, (**b**) VA-ROQS 5-fold cross-validation, (**c**) VA-ROQS center based validation.

**Figure 4:**
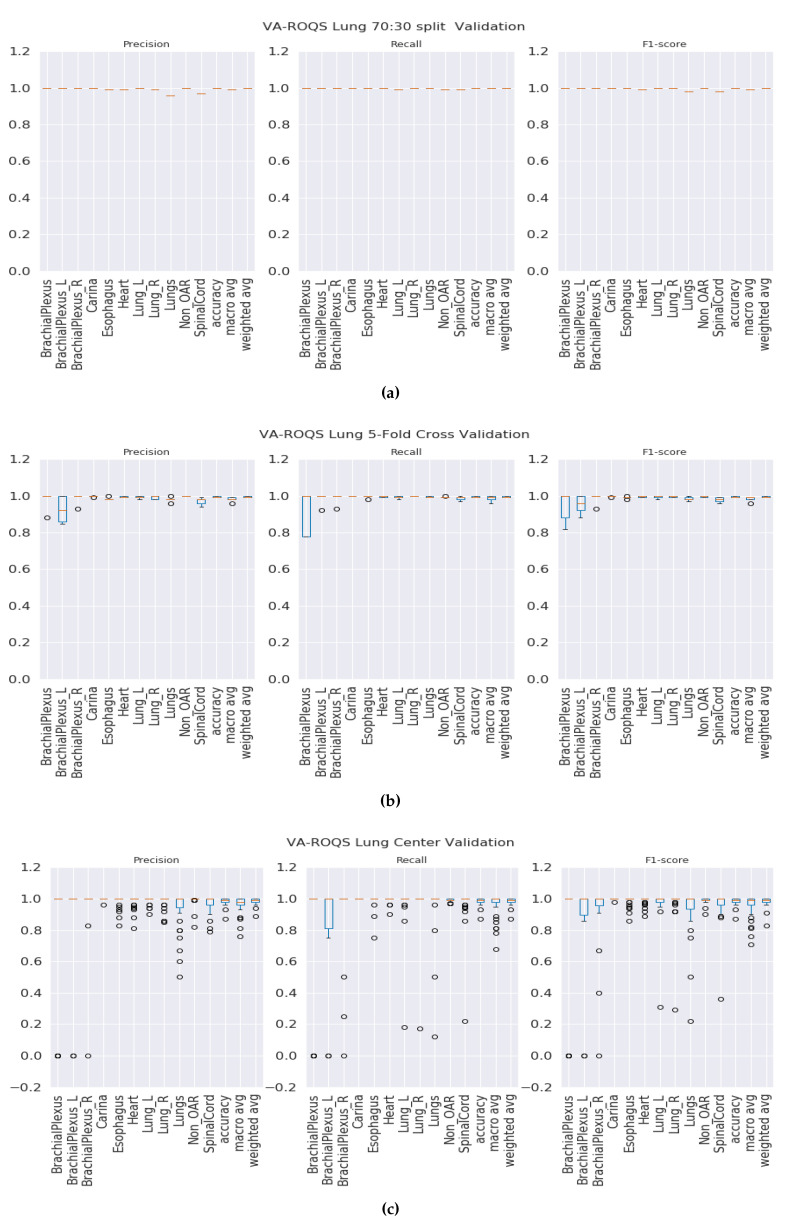
VA-ROQS lung dataset—cross-validation results: (**a**) VA-ROQS 70:30 split cross-validation (**b**) VA-ROQS 5-fold cross-validation (**c**) VA-ROQS center based validation.

**70:30 validation**: This validation type was chosen to test the model generalizability when data was split into 70% for training and 30% for testing. We split the data such that 70% of the patients from each center were under the training set and the rest of the patients from each center were under the testing set. We observed that our method was able to generalize well, and our model achieved overall macro-averaged F_1_ scores of 0.97 and 1.0 for prostate and lung datasets respectively. That indicates that our model was able to predict each label correctly. We also observed that our results were consistent across all classes regardless of class imbalance. Figure 3a and Figure 4a show the class-wise results for prostate and lung data.

***K*****-fold validation:** With this validation type we checked the performance on the complete dataset. Here, we split the data into K-folds using a K value of 5. We observed that the 5-fold cross-validation achieved overall macro-averaged F_1_ scores of 0.96 and 0.98 for prostate and lung datasets respectively. Excellent results from 5-fold validation indicates that our model was able to generalize the overall data and not just on some random split of the data. We also repeated the same process for 10-fold cross-validation and observed that the model achieved similar results with 0.96 and 0.99 macro-averaged F_1_ scores for prostate and lung respectively. We chose to present the 5-fold results here, and the 10-fold cross validation results are presented in the Figure S5 for the prostate and Figure S6 for the lung. It is important to see the consistent performance of each label in all folds. Figure 3b for the prostate and Figure 4b for the lung shows that our model has performed consistently well across all folds for each class and provided consistent performance.

**Center-based validation:** VA has 40 radiation therapy centers. Even though they all are under one VA management, we believe that there are some differences in their practices. Each center operates as an individual institution at the practice level. In order to test this hypothesis, we trained the model on the data from 39 centers and tested it on one center and repeated this process until all the centers had been tested. We observed that the model achieved 0.94 and 0.93 overall macro-average F_1_ scores for the prostate and lung respectively. Although the model performed well, the performance dropped by 2% for the prostate and around 6% for the lung. This indicates that our model has high performance, but the inherent variance in structure naming practices at the different VA centers caused the model to make some mistakes, which lead to a decrease in performance when compared to the first two validation types.

### 4.2. Test Results

**Figure 5:**
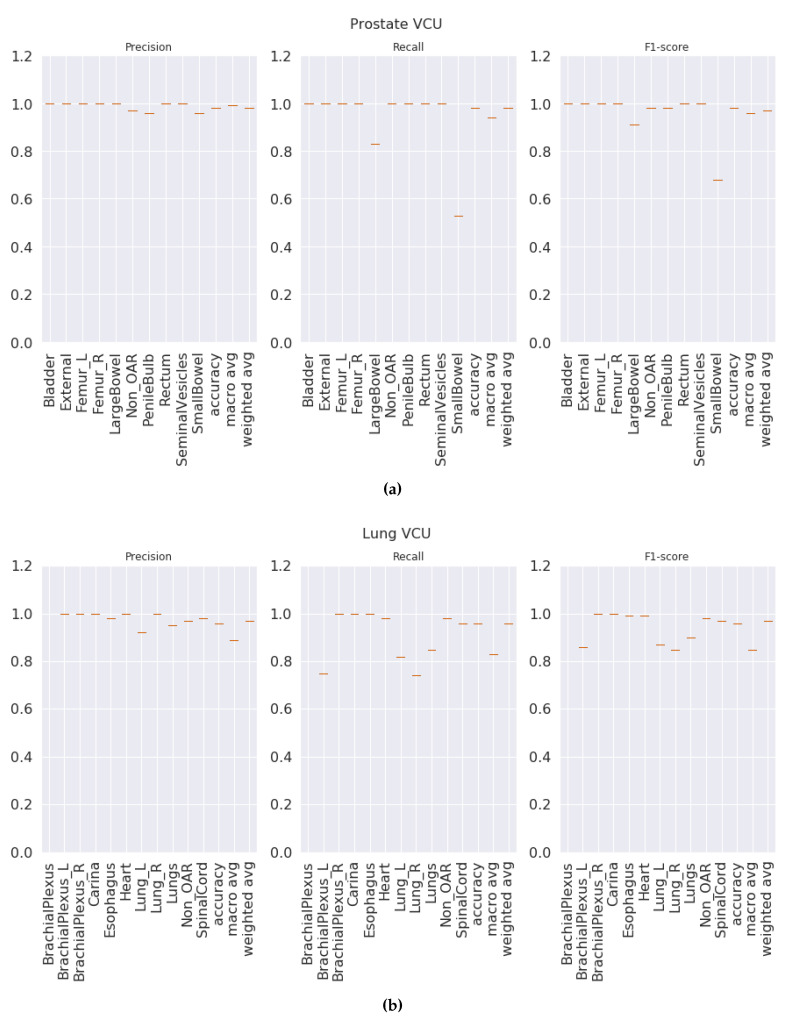
Test results (VCU dataset): (**a**) prostate, (**b**) lung.

Once the model is finalized after thorough validation methods, it is imperative to check the model’s performance on the unseen dataset. Here, the VCU dataset was used as a test set, which was never used in algorithm selection, model training, or validation. The final model was built with hyperparameters selected (see Section 3.7) on the entire VA-ROQS dataset. By using the VCU dataset as a test set, we were able to assess two aspects of our model. First, the model’s ability to generalize the on unseen data. Second, generalizability and transfer learning on a dataset from a different source. We observed that our model was able to predict the correct labels with high macro-averaged F_1_ scores of 0.94 and 0.86 for prostate and lung datasets, respectively. However, model performance dropped when compared to the model validation results, which indicates that although the model is robust, it is still affected by the change in the data source. We a observed drop in overall macro-average F_1_ score due to the one OAR label *BrachialPlexus*; VCU dataset did not have any OARs labeled *BrachialPlexus* but our model predicted the *BrachialPlexus_L* as *BrachialPlexus*. Even if the number of samples is very few, macro-averaged metrics give equal importance to all labels and penalize the overall score regardless of the number of instances of labels in the dataset. Figure 5a,b shows the class-wise results for prostate and lung data (see Tables S3 and S8 in supplementary material for individual class level results of prostate and lung). We suspect that it is because VCU is an academic medical center, unlike the VA, and hence the structure-naming practices at VCU differ to accommodate the needs of academic hospitals.

## 5. Discussion

The proposed radiotherapy structure name standardization methodology is system agnostic. Each of the validation types we presented on the VA-ROQS data demonstrates that our model is robust and works well to identify the correct TG-263 standardized names. We also tested our model with data from outside of the VA system (VCU dataset) which shows that our method works well for data from other institutions.

For the prostate RT structures, we observed that the majority of mistakes made by the model were in classifying *SmallBowel* and *LargeBowel*. This confusion is attributed to the fact that the same name can be used for both anatomical structures. In Table 6, we can see that “*bowel*” is used to label both *SmallBowel* and *LargeBowel*.

In the VCU Lung dataset validation, accuracy and macro-average F_1_ score dropped when compared to the 70:30 split validation. This drop was caused by the misclassification of the lung and brachial plexus related structures, as shown in Table 7.

### Error Analysis

**Figure 6:**
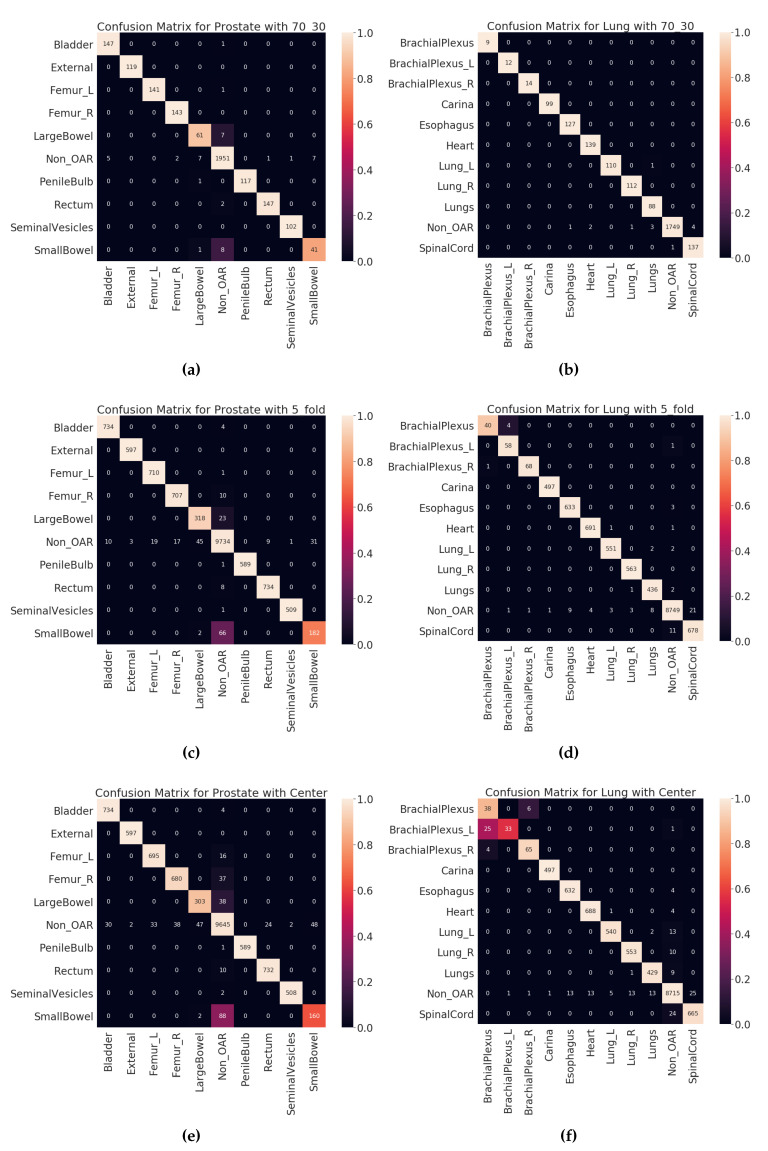
Validation set (VA-ROQS) confusion matrices of different validation types for both prostate and lung. (**a**) Prostate 70:30 split validation. (**b**) Lung 70:30 split validation. (**c**) Prostate 5-fold cross-validation. (**d**) Lung 5-fold cross-validation. (**e**) Prostate VA Center cross-validation. (**f**) Lung VA center cross-validation. Lighter color indicates better prediction. Diagonal indicates the correctly predicted labels.

**Figure 7:**
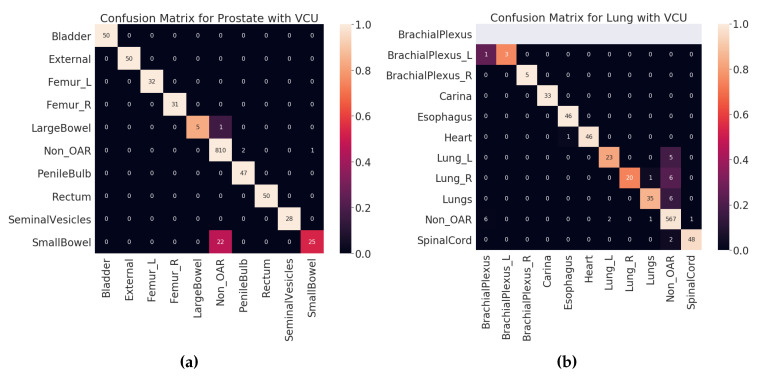
Test set (VCU) confusion matrices. (**a**) Prostate. (**b**) Lung. Lighter color indicates better prediction. Diagonal indicates the correctly predicted labels.

Figure 6 shows the confusion matrices for all validation types on validation dataset (VA-ROQS) and Figure 7 shows the confusion matrices for each test dataset (VCU). We performed error analysis on the test set to understand our model’s ability to generalize on unseen data. Error analysis provides the insights into the reasoning behind the failure of the model prediction. We need to look at the types of errors made by our model; to this effect, we divided misclassified predictions into three main categories.

- Type I: When the structure was OAR but predicted as Non_OAR.
- Type II: When the structure was OAR but predicted as the wrong OAR.
- Type III: When the structure was Non_OAR but predicted as OAR.

Type II and III errors are expensive errors when compared to the type I error, as they produce false-positive OAR. Below we will explain these errors based on each cancer type. Looking at the predicted and standard labels for physician-given names, we can infer that there is a pattern to errors for a few structures. Table 6 shows the errors made on VCU Prostate dataset. We observe that the majority of the errors come from type I. The major error was due to the lack of signal in the text label. Just looking at the structure name “bowel” and inferring the “SmallBowel” or “LargeBowel” structures is difficult even for experts.

In case of Lung, we see that there are many more type II an III errors made by the model. Table 7 shows all the errors made on the VCU Lung dataset. We can see that majority of the errors were made while predicting the structures related to the lungs (Lung_L, Lung_R, or Lungs) and brachial plexus. For lung-related structures we see that names containing numerical characters are most likely to be predicted as Non_OARs, as it is common for Non_OAR structures to contain numerical characters. For brachial plexus related structures, we can see that names containing “Plexus” are predicted as BrachialPlexus if there is no other information found to determine it as left or right BrachialPlexus. This also indicates the model errors due to the lack of signal in the input data. We also looked at the errors made by the model from holdout set (70:30 split) validation results (see Tables S13 and S14 for prostate and lung errors respectively). We observed a similar patterns of errors for the prostate; the major confusion is between “SmallBowel” and “LargeBowel”.

Our work differs in many ways when compared to the most recent proposed approaches in the research community. Schuler et al. reported that their approach resulted in a 99% relabel rate [4], but it requires the mappings from the domain expert from the same institute where data are collected. In contrast, our method provides the same success rate with the added advantage of working on arbitrary physician-given names from multiple institutes. Our work is scalable and generalizable to the external dataset. Two other works proposed machine-learning-based structure name standardization using geometric information [7,8]; both of those projects reported high accuracy. However, both of them did not use all the structures; instead they used only OARs. Our approach takes all possible structures into account and hence will work on real-world clinical datasets. However, due to the aforementioned limitations of the related work, it is not possible to perform a direct comparison between the accuracies from our approach and those from related work. It should also be noted that our proposed approach is the very first text mining based method to automatically standardize arbitrary structure names from the DICOM dataset.

## 6. Limitations and Future Work

Although a very high macro-average F1-score was achieved, we observed that our model made minor mistakes in identifying the correct TG-263 labels on the VCU dataset. To correct this issue, we plan to extend this work in two ways.

First, the fastText algorithm provides the probability of each predicted class. The probability of a class can be inferred as the model’s confidence in its prediction. By default, the model selects the class with the highest probability as it is prediction. However, this default setting generated high false positives. For example, there are nine standard structure names to choose from prostate data. If one class has 0.2 probability and the remaining is distributed rest of the classes, then the class with 0.2 probability is selected as a predicted class. It shows that the model has low confidence in its prediction and is most likely to provide a false-positive result. To avoid these false-positive predictions, we can accept the prediction only if it is above a certain threshold. Selecting the class above some high threshold will increase the model’s precision but will decrease the recall. It is crucial to have high precision and reasonably low recall; in the real world, false positives are more expensive than false negatives. It is vital to predict the correct labels in the structure name standardization process than being able to even predict all labels. False positives (wrong OAR labels) can hurt all downstream analyses. Hence, in the future, we will flag the low probability predictions to be verified by human experts, and these human-corrected predictions can then be used to retrain the model.

Secondly, just using physician-given names to predict the standard names has provided excellent results. However, we observed in some cases, just physician-given names are not enough to predict the standard label. For example, “Bowel” has been used by physicians to label SmallBowel and LargeBowel. It is clear from the example that physician-given names are not enough to build a highly accurate model for all the classes. We expect that image-based features will best augment the word-embedding-based features, which by themselves worked well, as demonstrated in this work. In the case where the combined model (word embedding with geometric information) is not enough, we plan to extend this model by further incorporating dose and volume data from the patient data to serve as additional features for consideration.

Our proposed model has three limitations. Firstly, we are only predicting the identities of the OARs and labeling them with standard names. However, the target (tumors) and PRVs are important structures and identifying and labeling them is also crucial for treatment delivery quality assessment. Secondly, we demonstrated that we can train on data from one institution and predict data from another. Our model is also language dependent, as it was trained only on structure names written in English. We believe the model pipeline will work for any language, but inter language models are only possible if they are trained on a mixture of languages. Thirdly, the ML pipeline from data preprocessing to prediction works as a standalone system. In the future, we plan to create a seamless enterprise informatics platform that can automatically collect data from the treatment planning systems and perform automatic structure name standardization on retrospective data.

## 7. Conclusions

In this paper, we presented a machine learning approach to standardize the radiotherapy structure names. We observed that the fastText algorithm works best when compared to other feature weighting and classification algorithms. Our method was evaluated with the data from 40 VA radiotherapy centers and tested on an external dataset from VCU. We demonstrated that our method works well on multiple disease sites and is also generalizable. To the best of our knowledge, this is the first and the only model using the physician-given name to predict the TG-263 standard name using NLP and machine-learning-based methods. We also observed that our approach fails in certain conditions, when enough information is not available for the model to infer the correct label. Our approach can be augmented with other available information, such as geometric information of structures. We believe that the proposed structure names standardization methods can help with big data analytics in the radiation therapy domain using population-derived datasets, including standardization of the treatment planning process, clinical decision support systems, treatment quality improvement programs, and hypothesis-driven clinical research.

## Figures and Tables

**Table 1:** Lung structure type distribution in VA-ROQS and VCU datasets.

	VA-ROQS Non Standard Name		VCU Non Standard Name	
Standard Name	Total Count	Unique Count	Total Count	Unique Count
Brachial_Plexus	44	11	0	0
Brachial_Plexus_L	59	14	4	5
Brachial_Plexus_R	69	23	5	3
Carina	497	7	33	2
Esophagus	636	28	46	4
Heart	693	21	47	2
Lung_L	553	46	28	10
Lung_R	563	46	27	10
Lungs	439	39	41	10
Non_OAR	8800	3701	577	259
SpinalCord	689	37	50	7
Total	13,044	3973	858	309

**Table 2:** Prostate structure type distribution in VA-ROQS and VCU datasets.

	VA-ROQS Non Standard Name		VCU Non Standard Name	
Standard Name	Total Count	Unique Count	Total Count	Unique Count
SmallBowel	250	40	47	7
LargeBowel	341	33	6	2
Femur_R	717	62	31	14
Femur_L	711	59	32	16
Rectum	742	14	50	3
Bladder	738	10	50	3
External	597	5	50	1
SeminalVesicles	510	50	28	8
PenileBulb	590	33	47	12
Non_OAR	9869	2886	813	425
Total	15,065	3195	1154	491

**Table 3:** Examples of physician-given names of structures from 40 VHA-ROQS centers. These are some examples from all the names given by physicians.

TG-263 Standard Name	Examples of Physician-Given Names
LargeBowel	Colon_Sigmoid, BOWEL LARGE, Bowel, sigmoid colon,
	Bowel_LG, SIGMOID_COLON, colon, Sigmoid OAR,
	Bowel NOS, large bowl, Sigmoid AZ, large bowel,
	Lg bowel, LG BOWEL, COLON_partial, LargeBowel,
	Sigmoid-AZ, Bowel Large, Rectosigmoid, Sigmoid Colon,
	LARGE BOWEL, SIGMOID08JUN16
Femur_L	FEMORAL LT, Femur_L, LFH, Femur_LT,
	Femoral Head Lt, Femoral Head_Lt, Lt Fem Head,
	FEMUR_L, left_femhead, Femur L,
	L_FEM HEAD, Lt Femur, Femur_Head_L,
	Hip Left, Femur-Lt, Lt Femoral Head,
	Fem hd neck Lt, Lt Hip, lt fem head,
	Femoral Lt, Femoral Head L, FEM HEAD LT,
	L Fem Hd, Femur Left, Femur l.,
	lt femoral hd, Left Femoral head JPC

**Table 4:** fastText hyperparameters and values tested for tuning the model.

Parameter	Name	Best Value	Values Tested	Description
*epoch*	number of epochs	50	5, 10, 15, 20, 25, 35, 45, 50	This parameter is used to determine the number of times a model will see the entire dataset
*lr*	learning rate	1.0	0.05, 0.1, 0.25, 0.5, 0.7, 1.0	This determines the step size taken at each iteration while moving toward a minimum of loss function
*minn*	minnum length of char ngram	2	2	minimum length of subword used to build word vector
*maxn*	maximum length of char ngram	6	3, 4, 5, 6	maximum length of subword used to build word vector
*wordNgrams*	maximum length of char ngram	2	2	Along with unique terms consecutive n-terms word vectors are generated
*dim*	size of the word vector	300	100, 150, 200, 250, 300	In ML context word vectors are numerical representations of word. dim indicates the length of the representation
*ws*	size of the context window	3	3, 4, 5, 6	Word vectors are build in such a way that it can predict the neighboring words in given text. It helps to encode the semantics of word. Window size indicates the range of words to predict.
*loss*	loss function	softmax	ns, hs, ova, softmax	A loss function is a measure of how good a prediction model does in terms of being able to predict the expected outcome.

**Table 5:** Disease specific macro-averaged precision, recall, F_1_ score, and overall accuracy for validation and test sets.

Evaluation Type	Disease	Validation Type	Precision	Recall	F_1_ score	Accuracy
Validation (VA-ROQS)	Prostate	70:30	0.97	0.97	0.97	0.99
		5-fold	0.96	0.96	0.96	0.98
		10-fold	0.96	0.97	0.96	0.98
		VA Center	0.94	0.94	0.94	0.97
	Lung	70:30	1.00	0.99	0.99	1.00
		5-fold	0.98	0.98	0.98	0.99
		10-fold	0.99	0.99	0.99	0.99
		VA Center	0.93	0.93	0.93	0.99
Test (VCU)	Prostate	-	0.94	0.99	0.96	0.98
	Lung	-	0.83	0.89	0.86	0.96

**Table 6:** Error analysis of VCU dataset prostate structure.

Error Type	Physician Given Name	TG-263 Name Standard Name	Predicted Name	Count
Type I	bowel	LargeBowel	Non_OAR	1
	bowel	SmallBowel	Non_OAR	22
Type II	nonptvpenilebulb	Non_OAR	PenileBulb	2
	small bowel	Non_OAR	SmallBowel	1

**Table 7:** Error analysis of VCU dataset lung structure names.

Error Type	Physician Given Name	TG-263 Standard Name	Predicted Name	Count
Type I	bilatlungs	Lungs	Non_OAR	5
	ptv	Lungs	Non_OAR	1
	lung-l	Lung_L	Non_OAR	1
	lung_l1	Lung_L	Non_OAR	4
	lung-r	Lung_R	Non_OAR	2
	lung_r1	Lung_R	Non_OAR	4
	spinal column	SpinalCord	Non_OAR	1
	spine	SpinalCord	Non_OAR	1
Type II	brachial_plexus	BrachialPlexus_L	BrachialPlexus	1
	esophagus	Heart	Esophagus	1
	lung	Lung_R	Lungs	1
Type III	ipsi_lung	Non_OAR	Lung_L	1
	left lung	Non_OAR	Lung_L	1
	brachial plexus	Non_OAR	BrachialPlexus	1
	brachial_plexus	Non_OAR	BrachialPlexus	2
	lung	Non_OAR	Lungs	1
	plexus	Non_OAR	BrachialPlexus	3
	t7 cord	Non_OAR	SpinalCord	1

## Supplementary Materials

The following are available online at https://www.mdpi.com/2227-9032/8/2/120/s1, Figure S1: Radiotherapy Structure name distribution per center for Prostate cancer in the VA-ROQS dataset; Figure S2: Radiotherapy Structure names distribution per center for Lung cancer patients in the VA-ROQS dataset; Figure S3: Hyperaparameter Tuning of fasttext for VA-ROQS Prostate cancer dataset; Figure S4: Hyperaparameter Tuning of fasttext for VA-ROQS Lung cancer dataset; Figure S5: VA-ROQS Prostate 10 fold cross-validation results; Figure S6: VA-ROQS Lung 10 fold cross-validation results; Table S1: Initial Model Selection Results for VA-ROQS Prostate datasets; Table S2: Initial Model Selection Results for VA-ROQS Lung datasets; Table S3: VCU Test Set results of Prostate structures; Table S4: VA-ROQS dataset 70:30 validation results for Prostate structures; Table S5: VA-ROQS Prostate dataset 5 fold validation results; Table S6: VA-ROQS Prostate dataset 10 fold validation; Table S7: VA-ROQS Prostate Center validation results; Table S8: VCU Test Set results of Lung structures; Table S9: VA-ROQS Lung dataset 70:30 validation results; Table S10: VA-ROQS Lung dataset Center validation results; Table S11: VA-ROQS Lung dataset 5 fold validation results; Table S12: VA-ROQS Lung dataset 10 fold Validation results; Table S13: Error analysis of VA prostate structure names with 70:30 split validation; Table S14: Error analysis of VA-ROQS dataset Lung structure names with 70:30 validation.

## Abbreviations

The following abbreviations are used in this manuscript:

AAPM	American Association of Physicists in Medicine
ASTRO	American Society for Radiation Oncology
TG-263	Task Group-263
RT	Radiotherapy
ROQS	Radiation Oncology Quality Surveillance Program
VCU	Virginia Commonwealth University
VA	Veterans Affairs
VHA	Veterans Health Administration
OAR	Organs-at-risk
Non_OAR	Non organs-at-risk
CT	Computed tomography
MRI	Magnetic resonance imaging
PRV	Planning organs-at-risk volume
QA	Quality assurance
NLP	Natural language processing
ML	Machine learning
