# Supplementary material for: Integrated Natural Language Processing and Machine Learning Models for Standardizing Radiotherapy Structure Names
Source: Healthcare (Basel). 2020 Apr 30;8(2):120. doi: 10.3390/healthcare8020120 (PMC7348919; doi:10.3390/healthcare8020120)
Supplement: Supplementary file 1 [file healthcare-08-00120-s001.pdf]

Supplementary Materials: Integrated Natural Language Processing and Machine Learning Models for Standardizing Radiotherapy Structure Names

Khajamoinuddin Syed [1]*, William Sleeman IV, [1,2], Kevin Ivey,[4], Michael Hagan [2,3], Jatinder Palta [2,3], Rishabh Kapoor [2,3], and Preetam Ghosh [1]

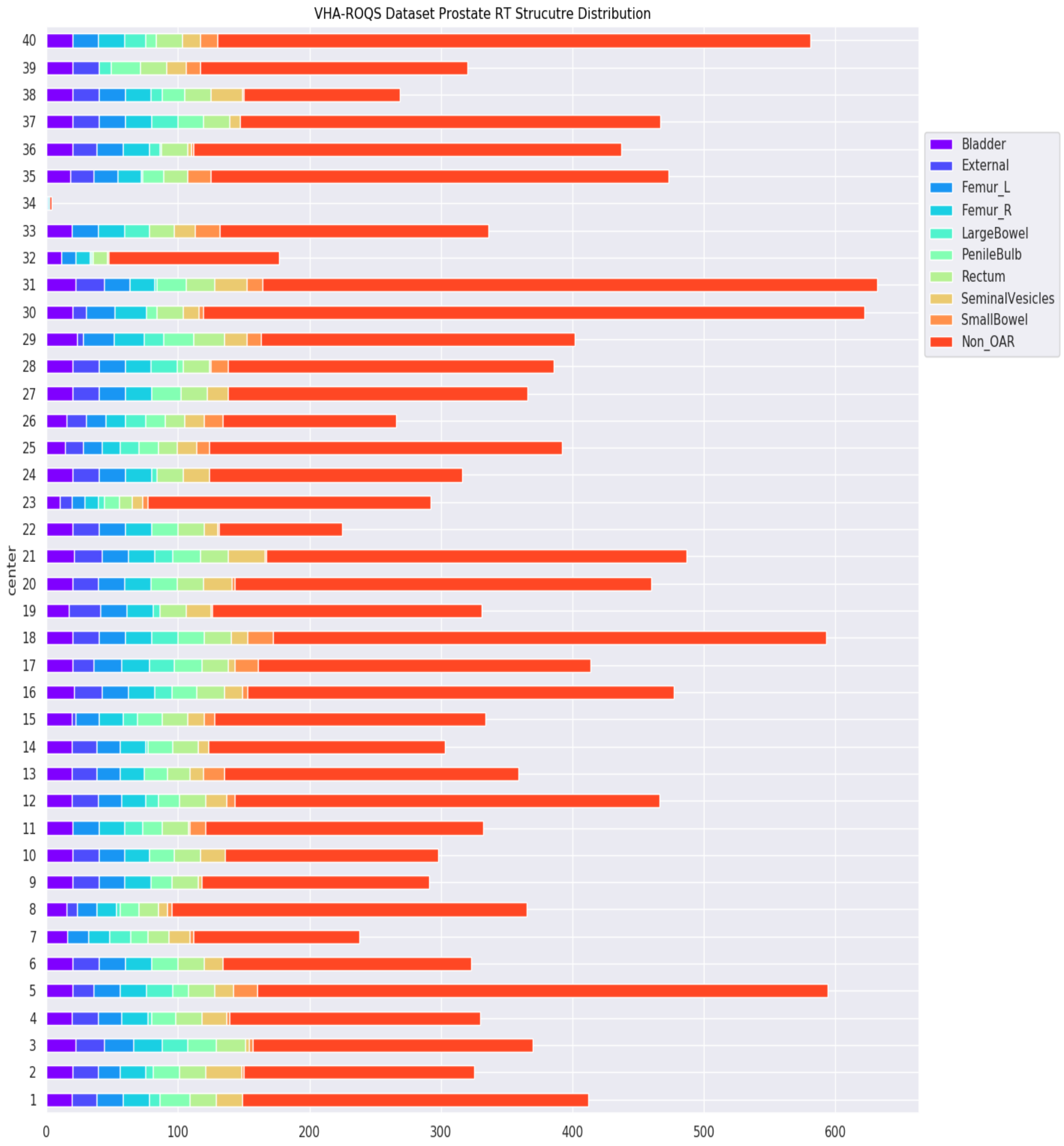

Figure S1. Radiotherapy Structure name distribution per center for Prostate cancer patients in the VA-ROQS dataset.

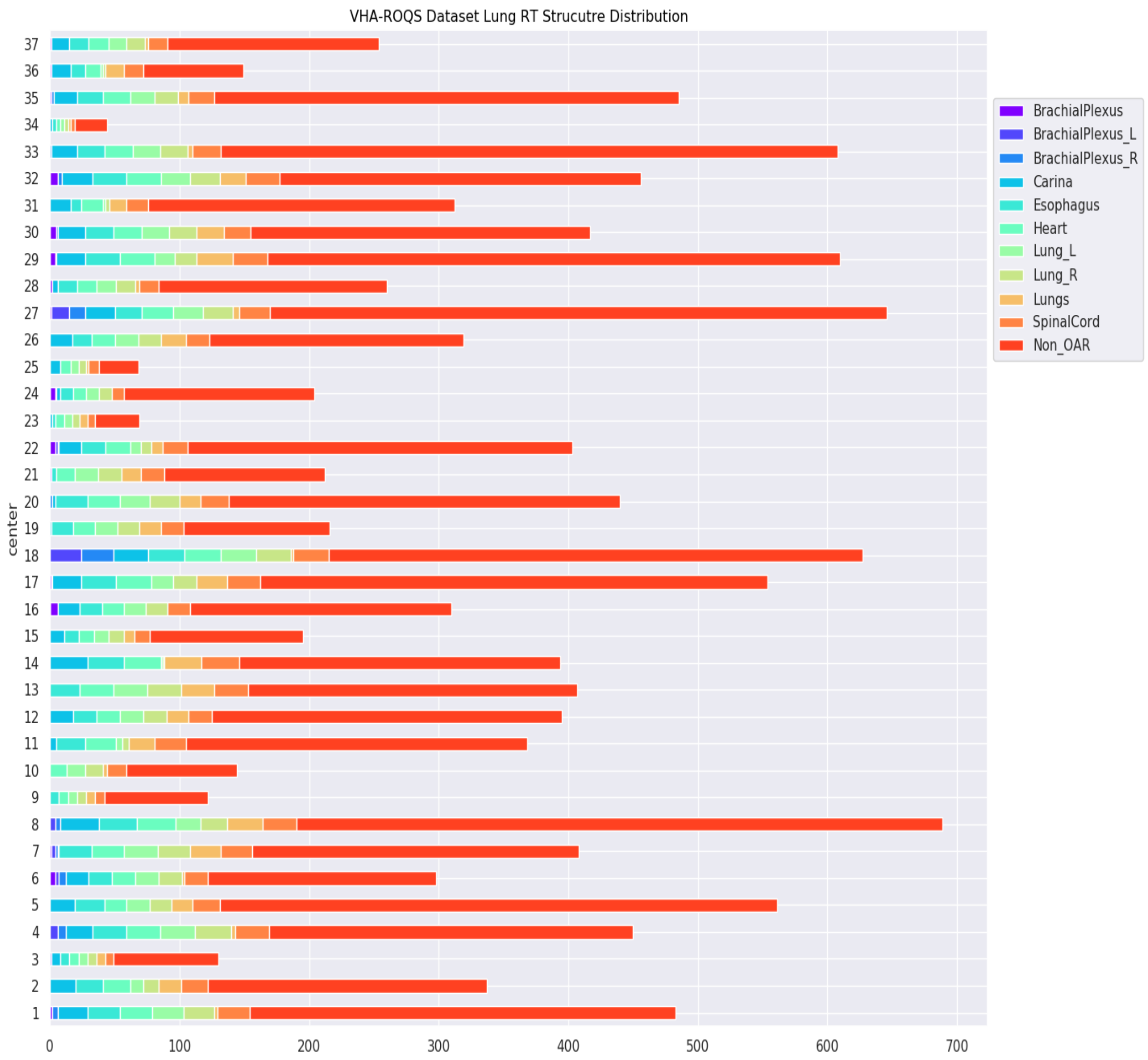

Figure S2. Radiotherapy Structure names distribution per center for Lung cancer patients in the VA-ROQS dataset.

Table S1. Initial Model Selection Results for VA-ROQS Prostate datasets

Features	Algorithm	Accuracy	Precision	Recall	F-Score
tp	SVM_RBF	0.99	0.96	0.97	**0.97**
	SVM_Linear	0.99	0.96	0.97	**0.97**
	Random_Forest	0.98	0.96	0.97	0.96
	Logistic_Regression	0.99	0.97	0.97	**0.97**
	KNeighbors	0.97	0.94	0.96	0.95
tc	SVM_RBF	0.99	0.96	0.97	**0.97**
	SVM_Linear	0.99	0.96	0.97	**0.97**
	Random_Forest	0.98	0.96	0.97	0.96
	Logistic_Regression	0.99	0.97	0.97	**0.97**
	KNeighbors	0.98	0.94	0.97	0.95
tf	SVM_RBF	0.99	0.96	0.97	**0.97**
	SVM_Linear	0.99	0.96	0.97	**0.97**
	Random_Forest	0.98	0.96	0.97	0.96
	Logistic_Regression	0.99	0.97	0.97	**0.97**
	KNeighbors	0.98	0.94	0.97	0.95
tf-idf	SVM_RBF	0.99	0.97	0.96	**0.97**
	SVM_Linear	0.99	0.97	0.97	**0.97**
	Random_Forest	0.99	0.96	0.97	**0.97**
	Logistic_Regression	0.98	0.97	0.96	0.96
	KNeighbors	0.98	0.95	0.97	0.96
Word-vectors	fastText	0.99	0.97	0.97	**0.97**

Table S2. Initial Model Selection Results for VA-ROQS Lung datasets

Features	Algorithm	Accuracy	Precision	Recall	F-Score
tp	SVM_RBF	0.99	0.95	0.92	0.93
	SVM_Linear	0.99	0.98	1.00	**0.99**
	Random_Forest	0.99	0.96	0.97	0.96
	Logistic_Regression	0.99	0.97	0.97	0.97
	KNeighbors	0.97	0.88	0.93	0.89
tc	SVM_RBF	0.99	0.95	0.92	0.93
	SVM_Linear	0.99	0.98	1.00	**0.99**
	Random_Forest	0.99	0.96	0.97	0.96
	Logistic_Regression	0.99	0.97	0.97	0.97
	KNeighbors	0.97	0.88	0.93	0.89
tf	SVM_RBF	0.99	0.95	0.92	0.93
	SVM_Linear	0.99	0.98	1.00	**0.99**
	Random_Forest	0.99	0.96	0.97	0.96
	Logistic_Regression	0.99	0.98	0.98	0.98
	KNeighbors	0.97	0.88	0.93	0.89
tf-idf	SVM_RBF	0.99	0.94	0.94	0.94
	SVM_Linear	0.99	0.93	0.93	0.92
	Random_Forest	0.99	0.96	0.97	0.96
	Logistic_Regression	0.99	0.94	0.90	0.92
	KNeighbors	0.98	0.89	0.92	0.90
Word-vectors	fastText	1.00	1.00	0.99	**0.99**

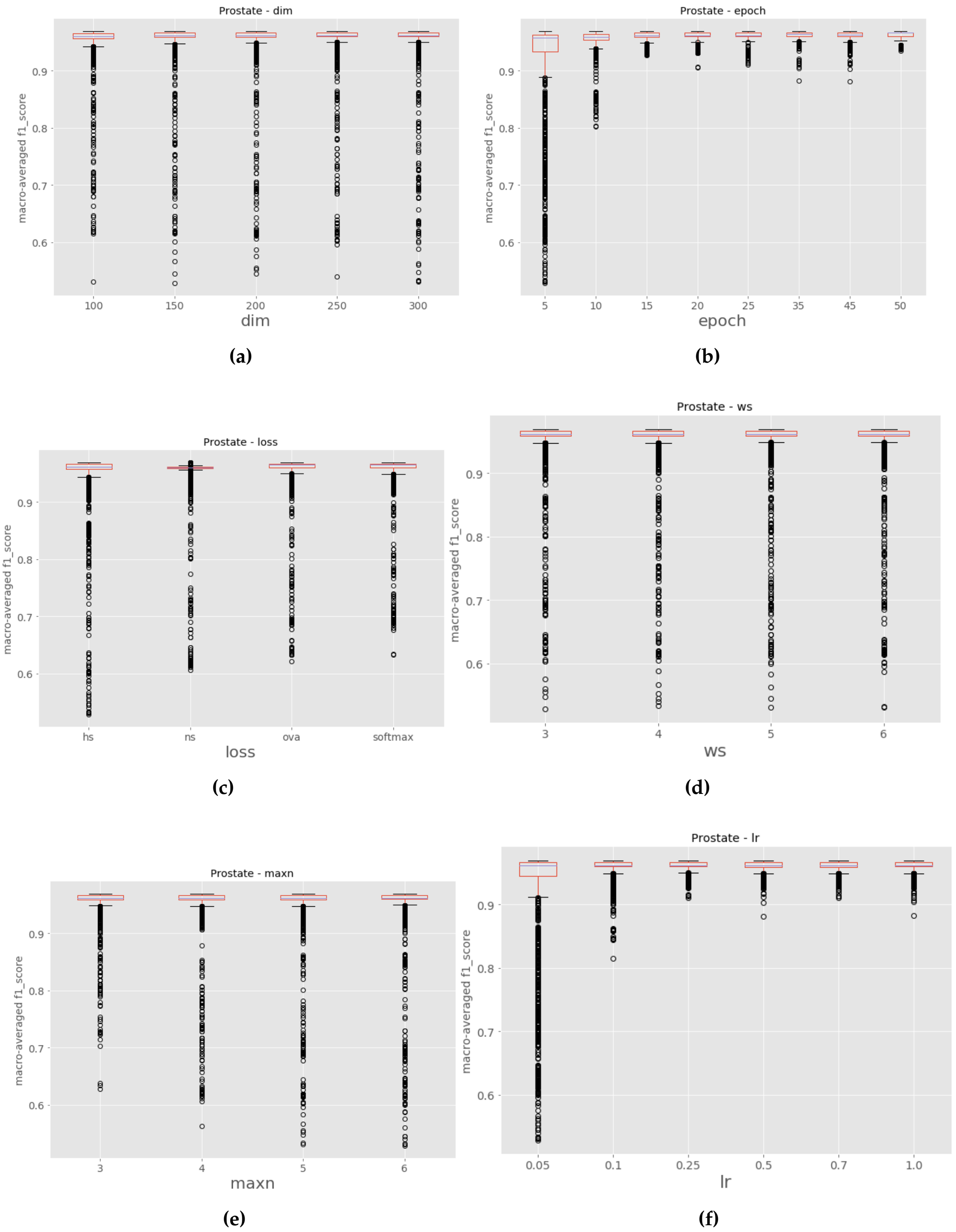

Figure S3. Hyperaparameter Tuning of fasttext for VA-ROQS Prostate cancer dataset. (a) dim: size of vector (b) epoch: number of times a model see's the all of the data while training, (c) loss, (d) ws: context window size (e) maxn: maximum length of character ngram (f) lr: learning rate

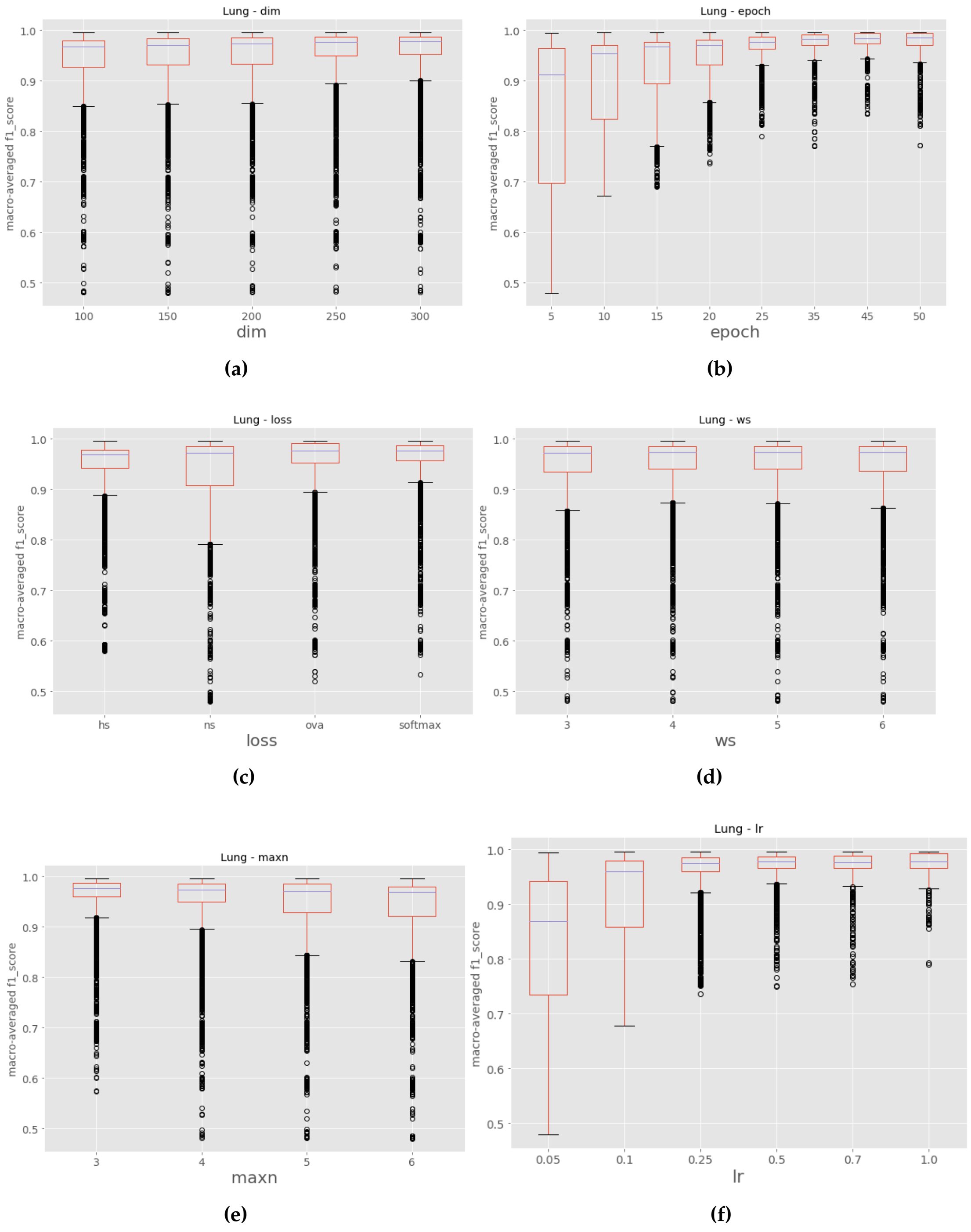

Figure S4. Hyperaparameter Tuning of fasttext for VA-ROQS Lung cancer dataset. (a) dim: size of vector (b) epoch: number of times a model see's the all of the data while training, (c) loss, (d)ws: context window size (e) maxn: maximum length of character ngram (f) lr: learning rate

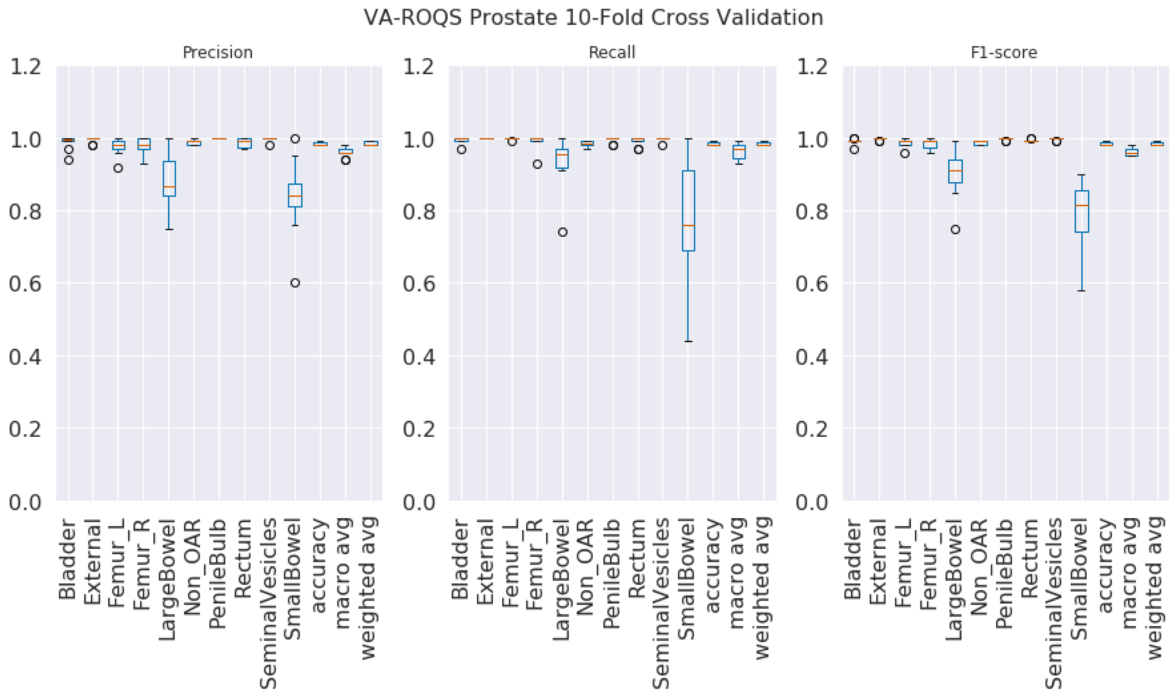

Figure S5. VA-ROQS Prostate 10 fold cross-validation results

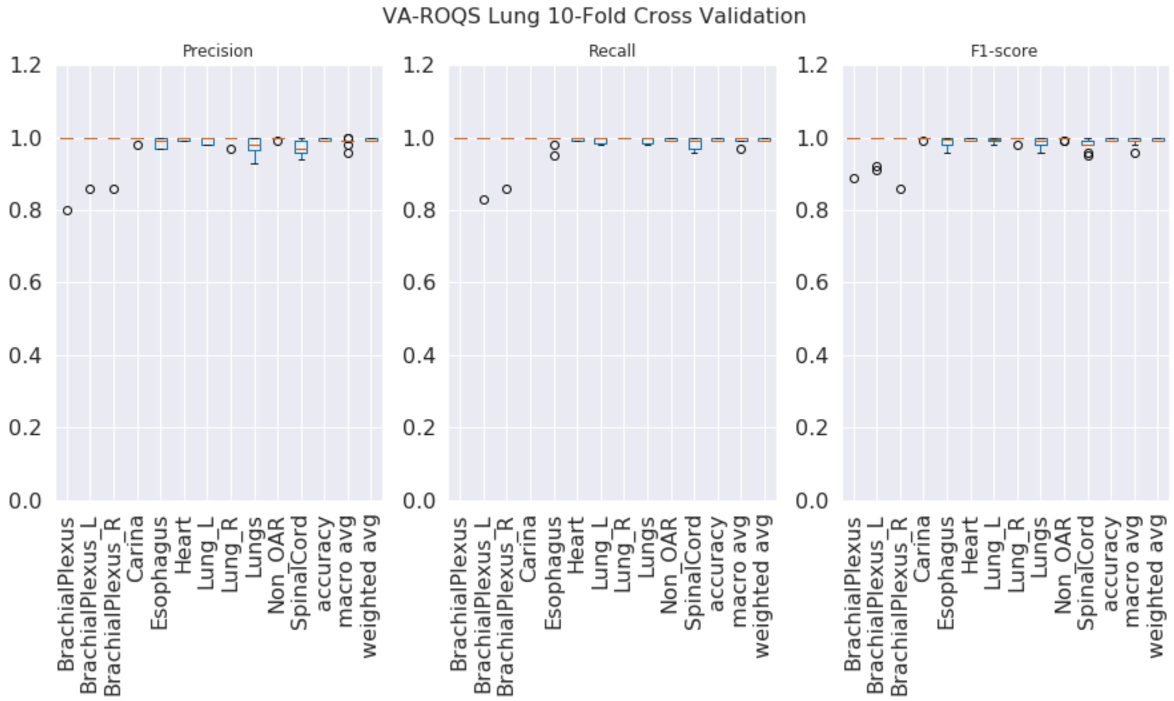

Figure S6. VA-ROQS Lung 10 fold cross-validation results

Table S3. VCU Test Set results of Prostate structures

Structure Name	Precision	Recall	F-Score	Support
Bladder	1.0	1.0	1.0	50
External	1.0	1.0	1.0	50
Femur_L	1.0	1.0	1.0	32
Femur_R	1.0	1.0	1.0	31
LargeBowel	0.83	1.0	0.91	5
Non_OAR	1.0	0.97	0.98	833
PenileBulb	1.0	0.96	0.98	49
Rectum	1.0	1.0	1.0	50
SeminalVesicles	1.0	1.0	1.0	28
SmallBowel	0.53	0.96	0.68	26
accuracy	0.98	0.98	0.98	1154
macro avg	0.94	0.99	0.96	1154
weighted avg	0.99	0.98	0.98	1154

Table S4. VA-ROQS dataset 70:30 validation results for Prostate structures

Structure Name	Precision	Recall	F-Score	Support
Bladder	0.99	0.97	0.98	152
External	1.0	1.0	1.0	119
Femur_L	0.99	1.0	1.0	141
Femur_R	1.0	0.99	0.99	145
LargeBowel	0.9	0.87	0.88	70
Non_OAR	0.99	0.99	0.99	1970
PenileBulb	0.99	1.0	1.0	117
Rectum	0.99	0.99	0.99	148
SeminalVesicles	1.0	0.99	1.0	103
SmallBowel	0.82	0.85	0.84	48
accuracy	0.99	0.99	0.99	3013
macro avg	0.97	0.97	0.97	3013
weighted avg	0.99	0.99	0.99	3013

Table S5. VA-ROQS Prostate dataset 5 fold validation results

Structure Name	Precision	Recall	F-Score	Support
Bladder	0.99	0.99	0.99	738
External	1.0	1.0	1.0	597
Femur_L	0.97	1.0	0.99	711
Femur_R	0.98	0.99	0.98	717
LargeBowel	0.87	0.93	0.9	341
Non_OAR	0.99	0.99	0.99	9869
PenileBulb	1.0	1.0	1.0	590
Rectum	0.99	0.99	0.99	742
SeminalVesicles	1.0	1.0	1.0	510
SmallBowel	0.85	0.73	0.79	250
accuracy	0.98	0.98	0.98	15065
macro avg	0.96	0.96	0.96	15065
weighted avg	0.98	0.98	0.98	15065

Table S6. VA-ROQS Prostate dataset 10 fold validation results

Structure Name	Precision	Recall	F-Score	Support
Bladder	0.99	0.99	0.99	738
External	1.0	1.0	1.0	597
Femur_L	0.97	1.0	0.99	711
Femur_R	0.98	0.99	0.98	717
LargeBowel	0.87	0.93	0.9	341
Non_OAR	0.99	0.99	0.99	9869
PenileBulb	1.0	1.0	1.0	590
Rectum	0.99	0.99	0.99	742
SeminalVesicles	1.0	1.0	1.0	510
SmallBowel	0.81	0.76	0.79	250
accuracy	0.98	0.98	0.98	15065
macro avg	0.96	0.97	0.96	15065
weighted avg	0.98	0.98	0.98	15065

Table S7. VA-ROQS Prostate Center validation results

Structure Name	Precision	Recall	F-Score	Support
Bladder	0.96	0.99	0.98	738
External	1.0	1.0	1.0	597
Femur_L	0.95	0.98	0.97	711
Femur_R	0.95	0.95	0.95	717
LargeBowel	0.86	0.89	0.87	341
Non_OAR	0.98	0.98	0.98	9869
PenileBulb	1.0	1.0	1.0	590
Rectum	0.97	0.99	0.98	742
SeminalVesicles	1.0	1.0	1.0	510
SmallBowel	0.77	0.64	0.7	250
accuracy	0.97	0.97	0.97	15065
macro avg	0.94	0.94	0.94	15065
weighted avg	0.97	0.97	0.97	15065

Table S8. VCU Test Set results of Lung structures

Structure Name	Precision	Recall	F-Score	Support
BrachialPlexus	0.0	0.0	0.0	7
BrachialPlexus_L	0.75	1.0	0.86	3
BrachialPlexus_R	1.0	1.0	1.0	5
Carina	1.0	1.0	1.0	33
Esophagus	1.0	0.98	0.99	47
Heart	0.98	1.0	0.99	46
Lung_L	0.82	0.92	0.87	25
Lung_R	0.74	1.0	0.85	20
Lungs	0.85	0.95	0.9	37
Non_OAR	0.98	0.97	0.98	586
SpinalCord	0.96	0.98	0.97	49
accuracy	0.96	0.96	0.96	858
macro avg	0.83	0.89	0.85	858
weighted avg	0.96	0.96	0.96	858

Table S9. VA-ROQS Lung dataset 70:30 validation results

Structure Name	Precision	Recall	F-Score	Support
BrachialPlexus	1.0	1.0	1.0	9
BrachialPlexus_L	1.0	1.0	1.0	12
BrachialPlexus_R	1.0	1.0	1.0	14
Carina	1.0	1.0	1.0	99
Esophagus	1.0	0.99	1.0	128
Heart	1.0	0.99	0.99	141
Lung_L	0.99	1.0	1.0	110
Lung_R	1.0	0.99	1.0	113
Lungs	1.0	0.96	0.98	92
Non_OAR	0.99	1.0	1.0	1750
SpinalCord	0.99	0.97	0.98	141
accuracy	1.0	1.0	1.0	2609
macro avg	1.0	0.99	0.99	2609
weighted avg	1.0	1.0	0.99	2609

Table S10. VA-ROQS Lung dataset Center validation results

Structure Name	Precision	Recall	F-Score	Support
BrachialPlexus	0.57	0.86	0.68	44
BrachialPlexus_L	0.97	0.56	0.71	59
BrachialPlexus_R	0.9	0.94	0.92	69
Carina	1.0	1.0	1.0	497
Esophagus	0.98	0.99	0.99	636
Heart	0.98	0.99	0.99	693
Lung_L	0.99	0.97	0.98	555
Lung_R	0.98	0.98	0.98	563
Lungs	0.97	0.98	0.97	439
Non_OAR	0.99	0.99	0.99	8800
SpinalCord	0.96	0.97	0.96	689
accuracy	0.99	0.99	0.99	13044
macro avg	0.94	0.93	0.93	13044
weighted avg	0.99	0.99	0.99	13044

Table S11. VA-ROQS Lung dataset 5 fold validation results

Structure Name	Precision	Recall	F-Score	Support
BrachialPlexus	0.98	0.91	0.94	44
BrachialPlexus_L	0.92	0.98	0.95	59
BrachialPlexus_R	0.99	0.99	0.99	69
Carina	1.0	1.0	1.0	497
Esophagus	0.99	1.0	0.99	636
Heart	0.99	1.0	1.0	693
Lung_L	0.99	0.99	0.99	555
Lung_R	0.99	1.0	1.0	563
Lungs	0.98	0.99	0.99	439
Non_OAR	1.0	0.99	1.0	8800
SpinalCord	0.97	0.98	0.98	689
accuracy	0.99	0.99	0.99	13044
macro avg	0.98	0.98	0.98	13044
weighted avg	0.99	0.99	0.99	13044

Table S12. VA-ROQS Lung dataset 10 fold Validation results

Structure Name	Precision	Recall	F-Score	Support
BrachialPlexus	0.98	1.0	0.99	44
BrachialPlexus_L	0.98	0.98	0.98	59
BrachialPlexus_R	0.99	0.99	0.99	69
Carina	1.0	1.0	1.0	497
Esophagus	0.99	0.99	0.99	636
Heart	0.99	1.0	0.99	693
Lung_L	0.99	0.99	0.99	555
Lung_R	1.0	1.0	1.0	563
Lungs	0.98	0.99	0.99	439
Non_OAR	1.0	0.99	1.0	8800
SpinalCord	0.97	0.98	0.98	689
accuracy	0.99	0.99	0.99	13044
macro avg	0.99	0.99	0.99	13044
weighted avg	0.99	0.99	0.99	13044

Table S13. Error analysis of VA prostate structure names with **70:30** split validation.

Error Type	Name In Dataset	TG-263 Name	Predicted	Count
Type-I	bowel	SmallBowel	Non_OAR	6
	bowel	LargeBowel	Non_OAR	5
	bowel (partial)	SmallBowel	Non_OAR	1
	bowel large	LargeBowel	Non_OAR	1
	bowel, large	LargeBowel	Non_OAR	1
	bowel-ptv_sigm	SmallBowel	Non_OAR	1
	fem hd neck l	Femur_L	Non_OAR	1
	p bulb control	PenileBulb	Non_OAR	1
	rectum_om	Rectum	Non_OAR	1
	rectum_wm	Rectum	Non_OAR	1
	bladder min	Bladder	Non_OAR	1
Type-II	sigmoid	SmallBowel	LargeBowel	1
Type-III	sigmoid	Non_OAR	LargeBowel	5
	small bowel	Non_OAR	SmallBowel	4
	sm bowel	Non_OAR	SmallBowel	3
	bladder, nos	Non_OAR	Bladder	3
	whole_rectum	Non_OAR	Rectum	1
	colon	Non_OAR	LargeBowel	1
	bladder1	Non_OAR	Bladder	1
	femoral head r	Non_OAR	Femur_R	1
	vesicle bed	Non_OAR	SeminalVesicles	1
	femur r	Non_OAR	Femur_R	1
	large bowel	Non_OAR	LargeBowel	1

Table S14. Error analysis of VA-ROQS dataset Lung structure names with **70:30** validation.

Error Type	Name In Dataset	TG-263 Name	Predicted	Count
Type I	total_lung	Lungs	Non_OAR	2
	spinalcanal	SpinalCord	Non_OAR	2
	esophagus-kl	Esophagus	Non_OAR	1
	cord_0	SpinalCord	Non_OAR	1
Type III	cord	Non_OAR	SpinalCord	1
	es	Non_OAR	Esophagus	1
	cord3	Non_OAR	SpinalCord	2
	l lung lymph	Non_OAR	Lung_L	1
	heart2	Non_OAR	Heart	1
